# Three cholecystokinin receptors differentially regulate reproduction, digestion, lipid metabolism and growth in medaka

**DOI:** 10.1530/JOE-25-0353

**Published:** 2026-04-15

**Authors:** Koji Murashita, Yu Sato, Yuichi Ozaki, Hazuki Yoshinaga, Ana S Gomes, Takeshi Yamamoto, Ayaka Senzui, Ivar Rønnestad

**Affiliations:** ^1^ Physiological Function Division, Aquaculture Research Department, Fisheries Technology Institute, Japan Fisheries Research and Education Agency, Tamaki, Watarai, Mie, Japan; ^2^ Faculty of Marine Science and Technology, Fukui Prefectural University, Katsumi, Obama, Fukui, Japan; ^3^ Institute of Marine Research, Tromsø, Norway; ^4^ Department of Biological Sciences, University of Bergen, Bergen, Norway

**Keywords:** cholecystokinin, receptor, digestion, appetite, growth, reproduction, lipid metabolism

## Abstract

Cholecystokinin (Cck) is a multifunctional peptide hormone involved in the regulation of digestion, appetite, and reproduction. In this study, we generated medaka (*Oryzias latipes*) knockout lines for three distinct Cck receptors (*cck1r*^−/−^, *cck2ra*^−/−^, and *cck2rb*^−/−^) and conducted phenotypic analysis to elucidate their physiological functions. The *cck1r*^−/−^ fish exhibited markedly reduced digestive enzyme secretion and growth remained impaired until 98 days post-hatching (dph), indicating an essential role for Cck1r in digestive regulation and early development. The *cck2ra*^−/−^ fish showed a slight reduction in reproductive capacity and a moderate increase in lipid accumulation, implicating its involvement in reproductive maturation and lipid metabolism. The *cck2rb*^−/−^ fish exhibited complete female infertility, pronounced lipid accumulation, and enhanced somatic growth after 126 dph, suggesting a central role for Cck2rb in regulating lipid metabolism, reproduction, and body size. Transcriptomic analysis further supported receptor-specific roles in lipid metabolism and growth regulation. These findings reveal novel functions of Cck receptors in lipid metabolism and somatic growth, extending beyond their established roles in digestion and reproduction. This study provides insights into endocrine–metabolic integration in vertebrates and represents the first functional characterization of all three Cck receptor subtypes within a single vertebrate model.

## Introduction

Cholecystokinin (Cck) is a multifunctional peptide hormone that plays a central role in regulating the digestive process and feeding behavior (1). In vertebrates, including teleost fish, Cck is characterized by a conserved C-terminal octapeptide (Cck-8) that retains biological activity across species. The *cck* gene is expressed in the gastrointestinal tract and brain, and its presence has been confirmed in a wide range of fish taxa (2). Cck-immunoreactive cells are localized in the intestinal epithelium and enteric nervous system, reflecting its dual role in endocrine and neuronal signaling (3).

Cck acts as a satiety signal, suppressing food intake through both central and peripheral mechanisms (4). Administration of sulfated CCK-8 has been shown to reduce feeding behavior in teleost species (5), whereas antagonists of CCK signaling increases feed intake (6). These findings support the conserved anorexigenic role of Cck in fish. Beyond appetite regulation, Cck facilitates digestion through modulation of gastric emptying, gastric acid secretion, gallbladder contraction, intestinal motility, and pancreatic digestive enzyme secretion (7).

The expression of the *cck* gene and circulating levels of Cck are regulated by feeding, nutritional state, and dietary composition. Protein and lipid intake generally upregulates *cck* expression (8) and Cck secretion (9), whereas fasting suppresses them (10). Genomic studies have revealed that teleosts possess multiple *cck* genes (11), with two or three paralogs identified in species such as spotted river puffer (12). These paralogs exhibit region-specific and/or species-specific expression patterns along the intestinal tract, suggesting functional diversification. Cck signaling may partially vary depending on ecological and physiological contexts.

Similarly, multiple *cck *receptor genes have been identified in fish including distinct paralogs of the Cck1 receptor (Cck1r) and Cck2 receptor (Cck2r) (13). In general, *cck1r* is predominantly expressed in the gastrointestinal tract and may mediate digestive functions, while *cck2r* is primarily localized in brain and is proposed to participate in central regulation of feeding and neuroendocrine signaling (7). The presence of multiple *cck *receptor genes with distinct tissue distribution patterns implies possible functional specialization; however, the extent and nature of this divergence remain poorly understood. Notably, recent findings have revealed that hypothalamic-derived Cck can stimulate the release of follicle-stimulating hormone (Fsh) from the pituitary via activation of the Cck2rb receptor subtype (14). This novel pathway suggests that Cck may serve as a metabolic–reproductive integrator, linking nutritional status to reproductive function.

Despite growing evidence for the diverse physiological roles of Cck ligands in teleosts, the specific functions of individual Cck receptor subtypes remain largely unexplored. Although their involvement in digestion, appetite regulation, and reproduction has been suggested, it is still unclear how each receptor contributes to these processes. In particular, the roles of distinct Cck receptors in sexual maturation, digestive regulation, and appetite control have not been systematically investigated. Moreover, their potential involvement in nutrient metabolism and other metabolic pathways remains entirely unknown. These physiological processes are closely linked to somatic growth, yet the impact of Cck receptor signaling on growth has not been investigated.

In this study, we conducted detailed functional analysis of three distinct Cck receptor subtypes in medaka (*Oryzias latipes*). By generating receptor-specific knockout (KO) lines and examining a wide range of physiological phenotypes, we aimed to elucidate how each receptor contributes to sexual maturation, digestion, appetite regulation, metabolic function, and somatic growth, thereby uncovering receptor-specific roles in these processes.

## Materials and methods

### Fish husbandry and ethics

Cab inbred strain medaka were used as the wild type (WT) in this study. The fish were maintained under laboratory standard conditions (26°C, 14 h light:10 h darkness cycle) and fed a commercial diet (Marubeni Nisshin Feed Co., Ltd, Japan) supplemented with *Artemia* nauplii to satiation twice daily. Medaka were anesthetized with 0.03% (W/V) tricaine methanesulfonate (MS-222, Sigma-Aldrich, USA) prior to sample collection. All animal experiments were carried out in accordance with the guidelines of the Institutional Animal Care and Use Committee of the Fisheries Technology Institute and approved by the Committee (approval IACUC-FTI no. 22013).

### Generation of the Cckr KO lines

Genome database annotations predicted three *cck *receptor genes in medaka: *cck1r* (ENSORLT00000008780.2), *cck2ra* (ENSORLT00000043095.1), and *cck2rb* (ENSORLT00000022492.2). The open reading frames of these receptors were PCR-amplified from cDNA, cloned, and sequenced using the WT strain fish. The primer pairs used are listed in Table 1.

**Table 1:** Primers used in the present study.

Target gene	Sequence (5′–3′)	Use
*cck1r*	ATG​GGG​GAG​TCG​TTT​ACC​ACC​ATC​A	Cloning
	TCA​GTT​GTA​GGT​AAA​GCG​AGT​GCT​C	
*cck2ra*	ATG​GAG​AGG​GTG​GCG​GGA​AAC​GCC​A	Cloning
	TCA​GCA​GGT​GCC​AGC​GCT​GCT​GAC​G	
*cck2rb*	ATG​GAT​ACT​TTG​AGA​AAC​GAG​ACA​G	Cloning
	TCA​GCA​GTT​TCC​CAT​GGT​GCT​GAC	
*cck1r*	ATG​GGG​GAG​TCG​TTT​ACC​A	Tissue distribution
	ACG​GTC​CTG​TCG​ATG​TCT​TTG	
*cck2ra*	TTC​CCA​CGC​CTA​CCG​TGT​CA	Tissue distribution
	CAG​CAG​CAC​GTA​CCA​GGT​CT	
*cck2rb*	CGT​GAG​ACA​CAG​AGA​AGA​GGG	Tissue distribution
	CGC​ATT​CGC​TTG​TTG​ACA​GT	
*cck1r*	CCA​CCA​TCA​TCG​CTG​AAA​TTC​T	HRM 1st PCR
	TGC​TGC​TTT​ATT​GGT​GAC​ATT​TCC	
*cck1r*	CGT​CCG​GAT​TTT​CCT​CTA​CTG​C	HRM 2nd PCR
	CTC​ATC​CTC​CGG​TTC​CTC​AC	
*cck2ra*	CGT​TTC​GCA​TCC​TGC​TCT​ACT​C	HRM 1st PCR
	CCA​TCA​AGT​AGG​TGA​CAA​TCT​TGC	
*cck2ra*	TGG​TGC​TGA​CAC​TGA​ACA​AAC​G	HRM 2nd PCR
	AGT​ATG​TTG​GGG​ATG​AGC​GTG​A	
*cck2rb*	GGG​GGA​TTT​AAA​GAC​GGA​GAG​C	HRM 1st PCR
	TCA​CCT​TCT​CTA​GCC​CAC​TGG​A	
*cck2rb*	CTT​ACA​ACG​GGA​GCC​TGG​AGA​C	HRM 2nd PCR
	GTT​TGA​GCA​CGC​TCA​CCT​CTG​T	
*dmrt1bY*	CTG​CCG​GAA​CCA​CAG​CTT​GAA	Sex detection
	AAT​GTT​TGT​GAA​TCA​GAA​TTT​GGA​CA	
*agrp*	ATC​GAG​AGA​AGC​CGT​GTT​CC	qPCR
	GCA​GAC​GAG​TCC​TCC​TCG​TAG	
*pomc*	GGG​AAA​CCC​ATA​GGA​CGC​AA	qPCR
	AGA​TGT​CTG​CGA​ACC​CGA​AA	
Fshb*	TGG​AGA​TCT​ACA​GGC​GTC​GGT​AC	qPCR
	AGC​TCT​CCA​CAG​GGA​TGC​TG	
lhb*	TGC​CTT​ACC​AAG​GAC​CCC​TTG​ATG	qPCR
	AGG​GTA​TGT​GAC​TGA​CGG​ATC​CAC	
*nr1h3*	GAC​TTT​GCA​AAA​GCA​GGA​CTT​CAG	qPCR
	CAG​AAA​AGA​TGT​TGA​TGG​CGA​TG	
*pparab*	CCA​AAT​TCC​AGT​TTG​CCA​TGA​AG	qPCR
	AGGCCAGGCCGGTCTCCA	
*apoa1b*	TCC​ATA​CCA​GGA​GCT​CAA​GAC​CA	qPCR
	CAT​CAG​AAA​TGG​TGG​TGA​CAA​CG	
*apoba*	AAT​ATG​GCC​TTA​CTG​GCA​CTG​CT	qPCR
	TGA​CGC​TTT​GCT​CTT​AGC​TTG​TG	
*igf1*	TCT​CAC​TAC​TGC​TGT​GCG​TCC​TC	qPCR
	CGT​AGC​CCG​TTG​GTT​TAC​TGA​AA	

* Uehara *et al.* (14).

KO lines for *cck1r*, *cck2ra*, and *cck2rb* were generated using the CRISPR/Cas9 system based on the method of Ansai and Kinoshita (15). Targeting sequences for *cck1r* (5′-TTG​CCC​AGC​AAG​CTG​AGG-3′), *cck2ra* (5′-CCT​TCC​TGC​TGT​CCC​TCG-3′), and *cck2rb* (5′-AAG​CGT​GGA​CGG​GTT​CAC​GC-3′) were cloned into pDR274 (Addgene #42250, USA), followed by *in vitro* transcription of specific single-guide RNAs (sgRNAs). sgRNAs (50 ng/μL) and Cas9 protein (100 ng/μL; EnGen Cas9 NLS, New England Biolabs, USA) were co-injected into fertilized WT eggs.

F0 embryos were raised and crossed with WT fish to obtain F1 progeny. Mutations were confirmed by sequencing, and off-target effects were minimized through three generations of backcrossing. Homozygous KO lines were established via intercrossing. The *cck1r* and *cck2ra* mutants were fertile in both sexes, whereas *cck2rb* mutants were fertile only in males. Populations were expanded by sibling mating of homozygous (*cck1r* and *cck2ra*) and heterozygous (*cck2rb*) individuals. Genotyping was conducted using nested PCR-based high-resolution melting (HRM) analysis with target region-specific primer pairs (Table 1) and SYTO9 dye, on a LightCycler 96 System (Roche, Switzerland).

### Rearing experiment

Fertilized eggs collected from homozygous WT, *cck1r*, and *cck2ra* mutants (*n* = 200/line) and from *cck2rb* mutants (*n* = 400; homozygous male × heterozygous female) were distributed into Petri dishes (50 eggs/dish). Twenty-five of the newly hatched larvae were transferred from the Petri dishes to rearing tanks on the same day (four replicate tanks per line). *cck2rb* mutants were genotyped at 24 days post-hatching (dph) by fin-clipping and HRM analysis and classified into homozygous and heterozygous groups. Genetic sex was determined simultaneously by HRM targeting the *dmrt1bY* locus. Fish numbers were adjusted to 20/tank with a 1:1 sex ratio. Fish were fed commercial pellets gradually until they stopped feeding, ensuring no uneaten feed remained in tank. Feed intake was recorded daily. Tank volume was scaled with fish growth: 1.2 L (0–5 dph), 2.5 L (6–24 dph), 5.5 L (25–47 dph), and 11 L (48–208 dph).

After body weight measurement at 208 dph (following 24 h fasting), digestive enzyme secretion was compared between WT and KO lines. Fish were fasted for another 48 h and then fed 2.5% BW. Intestinal contents from three fish per tank were sampled 6 h post-feeding and stored at −80°C. At 215 dph, after 24 h fasting, two males and two females per tank were sampled for plasma lipid and vitellogenin analysis. Visceral fat, liver, and gonads were weighed; liver subsamples were stored at −80°C with or without RNAlater. Skeletal muscle was stored at −30°C. Hypothalamus and pituitary were preserved in RNAlater for 24 h and then stored at −80°C. Remaining fish were pooled by sex, homogenized, and stored at −30°C for lipid analysis (*n* = 4 tanks).

### Biochemical analysis

The activities of trypsin, chymotrypsin, lipase, and amylase in the intestinal content were assayed as described in Murashita *et al.* (16).

Lipid composition was analyzed in the whole body, skeletal muscle, liver, and plasma. The tissue samples were homogenized in 19× their volume of EtOH:diethyl ether (3:1). The homogenate was centrifuged at 8,000 *g* for 10 min, and the supernatant was used to determine lipid composition. The plasma sample was directly used for the assay. Concentrations of triglyceride and total cholesterol were measured using commercial kits (LabAssay Triglyceride and Cholesterol; Fujifilm Wako Pure Chemical, Japan). The plasma vitellogenin level was measured using the EnBio Medaka Vitellogenin ELISA Kit (Fujikura Kasei Co. Ltd, Japan).

### Real-time quantitative PCR (qPCR)

For tissue distribution analysis of the three *cckr* genes, whole brain, pituitary, eye, tongue, gill, skin, muscle, heart, gallbladder, liver, spleen, kidney, gonad, foregut, midgut, and hindgut were dissected from four male and four female WT fish. Additionally, pituitary glands were collected from 16 males and 16 females, with five glands pooled per sample.

Samples from fish used in the growth experiment were also analyzed. The genes examined included agouti-related protein (*agrp*) and pro-opiomelanocortin (*pomc*) in the hypothalamus, follicle-stimulating hormone beta-subuni*t* (*fshb*) and luteinizing hormone beta-subunit (*lhb*) in the pituitary, and nuclear receptor subfamily 1 group H member 3 (*nr1h3*), peroxisome proliferator-activated receptor alpha b (*pparab*), apolipoprotein a1b (*apoa1b*), apolipoprotein ba (*apoba*), and insulin-like growth factor 1 (*igf1*) in the liver.

Total RNA was extracted from the tissue samples using Sepasol-RNA1 Super G (Nakalai Tesque, Japan), and first-strand cDNA was synthesized using the Verso cDNA Synthesis Kit (Thermo Fisher Scientific, USA). Expression levels of target mRNAs were quantified using the LightCycler 96 System (Roche) with THUNDERBIRD SYBR qPCR Mix (TOYOBO, Japan). The primer pairs used for qPCR are listed in Table 1.

### Histology

Whole fish from each line were fixed in Davidson’s solution at three months post-hatching (*n* = 3). The fixed whole body was dehydrated using a series of ethanol solutions and embedded in paraffin. The embedded tissues were then cut transversely into 5 μm sections using a microtome (model RM2255; Leica Microsystems GmbH, Germany), and sections were stained with Mayer’s hematoxylin and eosin. The sections of gonads and liver were then observed under a microscope (model BX50; Olympus Corp., Japan).

### Transcriptomic analysis

Liver samples (*n* = 5/line) were collected at six months post-hatching, preserved in RNAlater, and stored at −80°C. Total RNA was extracted using the RNeasy Mini Kit (Qiagen, Netherlands), and samples with RQN ≥ 7.0 were sequenced by BGI (China) using the DNBSEQ platform (paired-end, 150 bp reads). Clean reads were mapped to the Japanese medaka Hd-rR reference genome (Ensembl release 85; https://ensembl.org) using STAR (17), and gene counts were obtained using featureCounts (18). Differentially expressed genes (DEGs) were identified using DESeq2 in iDEP.94 (19) with thresholds of ≥ twofold change and FDR *P* < 0.1. Functional enrichment analysis of DEGs was performed using Metascape (20), based on zebrafish orthologs and multiple annotation sources, including GO, KEGG, Reactome, and WikiPathways.

### Statistical analysis

All data are presented as means and standard deviations unless stated otherwise. The results were analyzed by one-way analysis of variance (ANOVA) using GraphPad Prism 9 (GraphPad Software, USA) unless stated otherwise. Differences between groups were assessed with Tukey’s multiple comparison test. A probability level of less than 0.05 was considered as significant.

## Results

### Generation of the *cckr* KO medaka line

**Figure 1:**
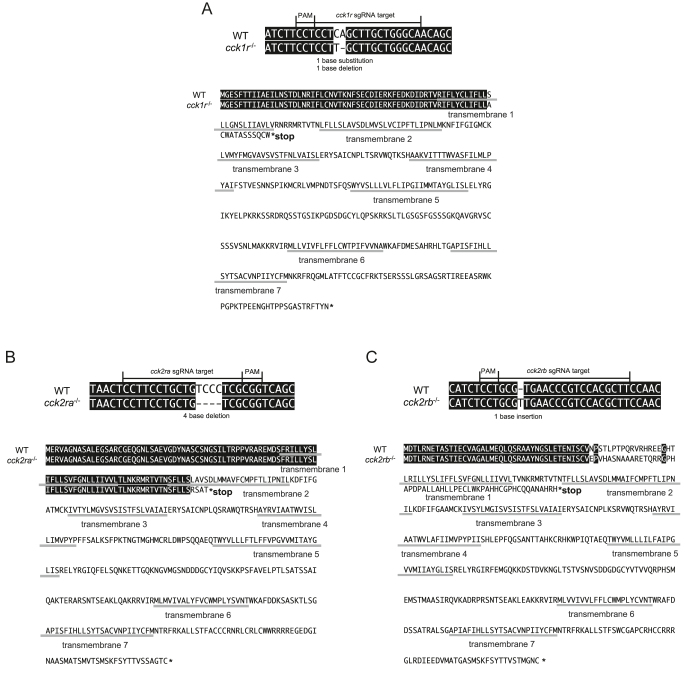
Generation of the *cckr* KO medaka. (A) CRISPR/Cas9 was used to target an 18 base pair (bp) region of the medaka *cck1r* gene, (B) an 18 bp region of *cck2ra*, and (C) a 20 bp region of *cck2rb*. Frameshift mutations in the *cckr* genes resulted in truncated proteins due to indels that disrupted the reading frame, leading to the generation of premature stop codons upstream of the first or second transmembrane domains. The transmembrane domains of the predicted amino acid sequences were identified using DeepTMHMM v1.0.

A targeted mutation was induced using CRISPR/Cas9 into each of the three medaka *cckr* genes (Fig. 1). A stable 1 bp substitution/1 bp deletion, 4 bp deletion, and 1 bp insertion occurring at residues 60, 93, and 42 resulted in a frameshift and premature stop codon after 12, 4, and 53 nonsense amino acids for *cck1r*, *cck2ra*, and *cck2rb*, respectively. The induced mutations were also confirmed in the cDNA sequence of the *cckr* mutants, indicating a loss of function.

### Tissue distribution

**Figure 2:**
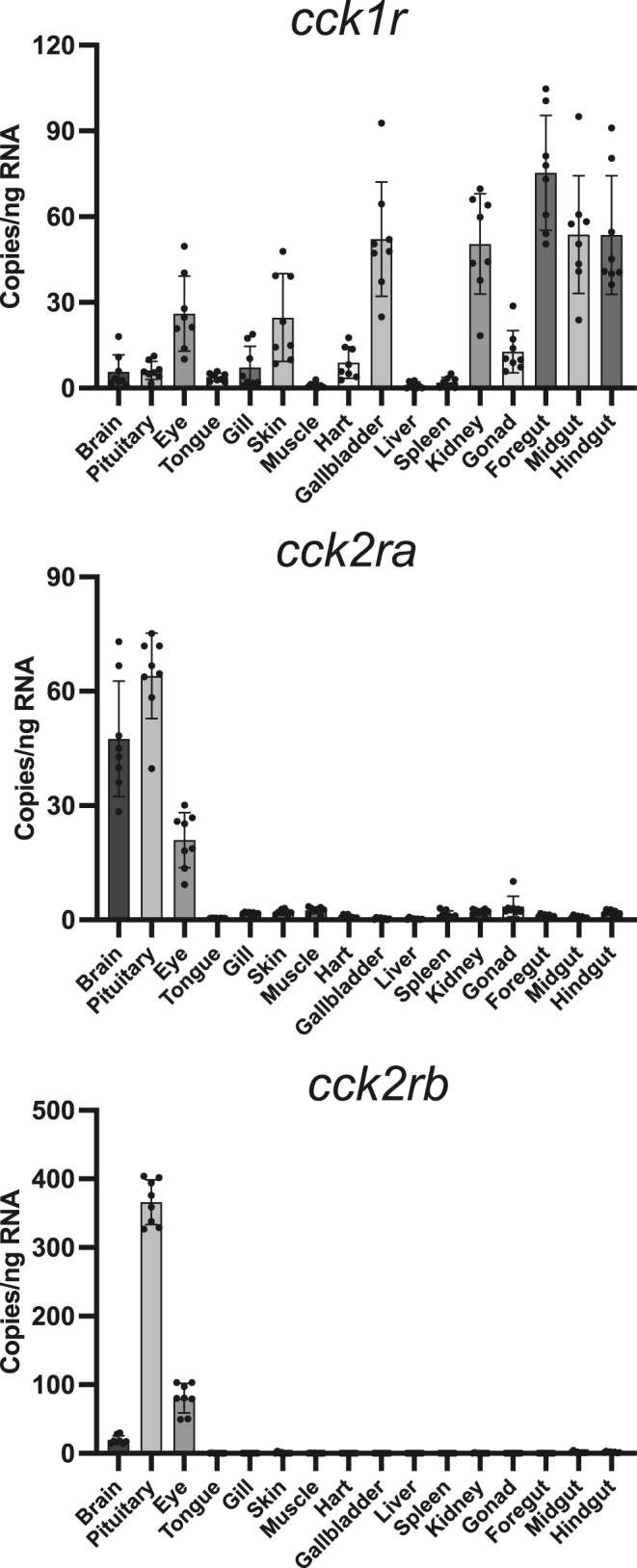
Tissue distribution of *cck1r*, *cck2ra*, and *cck2rb* mRNA in medaka. The data are presented as copy number means of four males and four females combined, since no sex differences were observed in tissue distributions (*n* = 8 fish).

The tissue distribution of the three *cckr* genes in medaka was analyzed (Fig. 2). *cck1r* was ubiquitously expressed, with high expression in digestion-related tissues, such as gallbladder, foregut, midgut, and hindgut. High expression levels of *cck2ra* were observed in brain, pituitary, and eye. *cck2rb* was mainly expressed in pituitary, followed by the eye and brain.

### Growth

**Figure 3:**
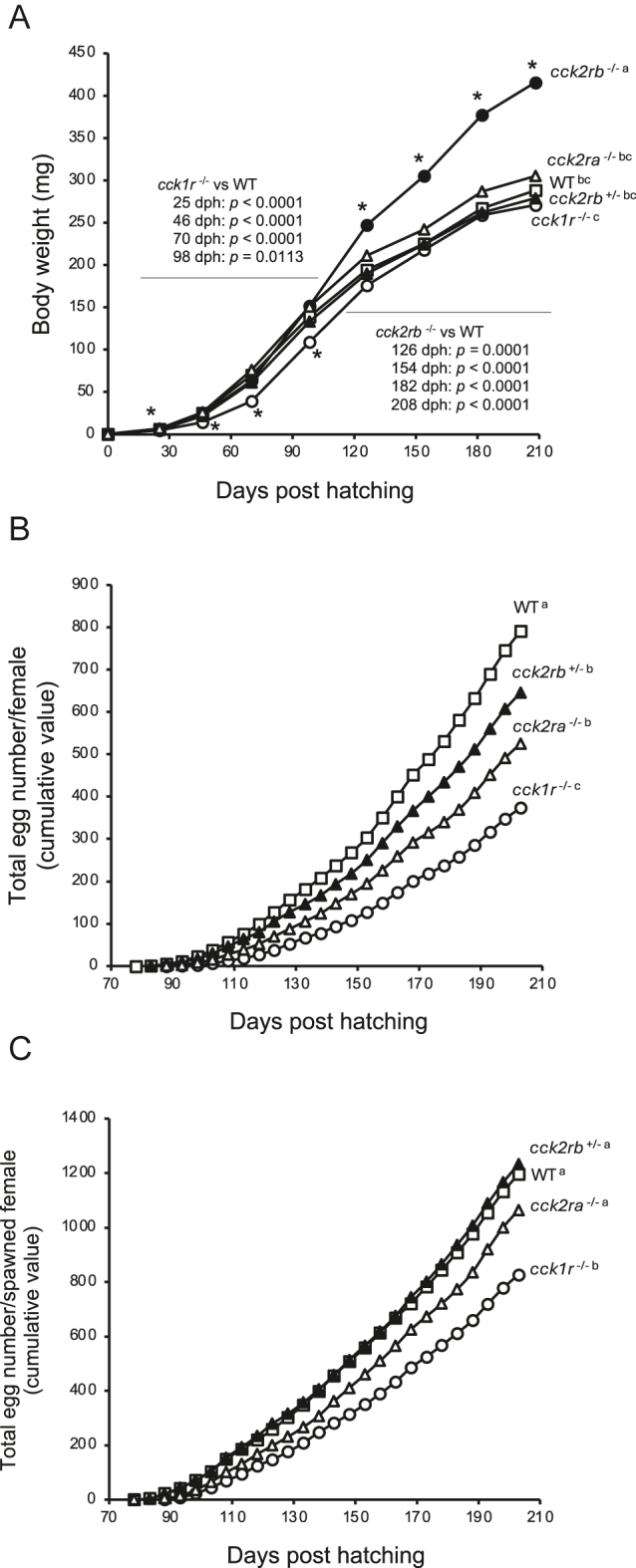
Growth and reproductive performance of *cckr* KO medaka during the trial. The open square, open circle, open triangle, closed circle, and closed triangle represent WT, *cck1r*^−/−^, *cck2ra*^−/−^, *cck2rb*^−/−^, and *cck2rb*^+/−^, respectively. Data points with different superscript letters are significantly different (*n* = 4, at tank level, *P* < 0.05). (A) Mean body weight of *cckr* KO medaka. An asterisk (*) indicates a statistically significant difference between *cck1r*^−/−^ and WT, or between *cck2rb*^−/−^ and WT. Body weights of fish were bulk weighed after a 24 h fasting period at 25, 46, 70, 98, 126, 154, 182, and 208 dph. The body weight of *cck1r*^−/−^ was significantly lower than that of the other lines at 25 (*P* < 0.0001), 46 (*P* < 0.0001), 70 (*P* < 0.0001), and 98 dph (*P* = 0.0113). From 126 dph onward, the body weight was comparable to WT (126 dph, *P* = 0.2575; 154 dph, *P* = 0.7894; 182 dph, *P* = 0.8786; 208 dph, *P* = 0.3787). The body weight of *cck2rb*^−/−^ was comparable to that of WT until 98 dph (*P* = 0.5719) but increased significantly thereafter, becoming significantly higher than that of the other lines at 126 (*P* = 0.0001), 154 (*P* < 0.0001), 182 (*P* < 0.0001), and 208 dph (*P* < 0.0001). In contrast, the body weight of *cck2rb*^+/−^ and *cck2ra*^−/−^ remained comparable to WT throughout the experimental period. (B) Total egg number/female (including non-spawning females in the calculation) (cumulative value) in *cck2ra*^−/−^ (*P* = 0.0005) and *cck2rb*^+/−^ (*P* = 0.0399) were lower than in WT, and that of *cck1r*^−/−^ was the lowest between the lines (*P* < 0.0001). (C) Total egg number/spawned female (cumulative value) in *cck2ra*^−/−^ (*P* = 0.1765) and *cck2rb*^+/−^ (*P* = 0.9242) were equivalent to that in WT, while that of *cck1r*^−/−^ was lower than in the other lines (*P* < 0.0001).

To compare growth and reproductive performance between WT and *cckr* KO mutants, fish from each line were reared for 208 days. The BW of *cck1r*^−/−^ was significantly lower than that of the other lines at early juvenile stage (25–98 dph, Fig. 3A and Table 2). The BW of *cck2rb*^−/−^ was comparable to that of WT until the juvenile stage but increased significantly thereafter, becoming significantly higher than that of the other lines at post-juvenile stage (126–208 dph). In contrast, the BW of *cck2rb*^+/−^ and *cck2ra*^−/−^ remained comparable to that of WT throughout the experimental period. The specific growth rate and the feed efficiency ratio of *cck1r*^−/−^ were significantly lower than those of WT during early juvenile stages (0–46 dp) but significantly higher during later stages (71–126 dp, Table 2). The specific growth rate of *cck2rb*^−/−^ was significantly higher than that of WT at 71–126 dph. In addition, the feed efficiency ratio of the *cck2rb*^−/−^ was consistently higher than that of WT throughout later period (71–208 dph). Feed intake in *cck2rb*^−/−^ was significantly lower than that of WT at post-juvenile stage (127–208 dph). Similarly, *cck2ra*^−/−^ exhibited relatively lower feed intake compared to WT at post-juvenile stage (99–208 dph). The condition factor of *cck1r*^−/−^ was lower than that of WT at early juvenile stage (46 and 70 dph, Table 3). In contrast, male *cck2rb*^−/−^ exhibited a significantly higher condition factor than the other lines at later periods (182 and 208 dph). A greater BW and standard length of both sexes of *cck2rb*^−/−^ were observed at a later period (126–208 dph).

**Table 2:** Growth performance of *cck *receptor KO lines reared for 208 days. Data are presented as mean ± SD (*n* = 4 tanks). Values with different letters are significantly different (*P* < 0.05).

Growth performance/dph	WT	*c* *ck1r* ^−/−^	*c* *ck2ra* ^−/−^	*c* *ck2rb* ^−/−^	*c* *ck2rb* ^+/−^	*P*
Body weight (mg)						
0	0.68 ± 0.02	0.68 ± 0.02	0.68 ± 0.02	0.68 ± 0.01	0.68 ± 0.01	0.9896
25	6.32 ± 0.27^ab^	4.68 ± 0.21^c^	6.70 ± 0.18^a^	5.91 ± 0.36^b^	5.73 ± 0.37^b^	<0.0001
46	25.1 ± 1.5^ab^	14.1 ± 2.9^c^	25.9 ± 0.8^a^	23.0 ± 1.1^ab^	21.9 ± 1.6^b^	<0.0001
70	70.3 ± 4.5^a^	39.1 ± 12.0^b^	75.6 ± 1.0^a^	63.6 ± 4.7^a^	61.3 ± 5.6^a^	<0.0001
98	139 ± 3^a^	109 ± 22^b^	151 ± 4^a^	151 ± 6^a^	133 ± 8^a^	0.0004
126	194 ± 7^bc^	176 ± 18^c^	211 ± 12^b^	247 ± 9^a^	190 ± 10^bc^	<0.0001
154	225 ± 10^bc^	218 ± 11^c^	242 ± 11^b^	305 ± 7^a^	224 ± 6^bc^	<0.0001
182	267 ± 13^bc^	259 ± 4^c^	287 ± 16^b^	377 ± 17^a^	262 ± 8^bc^	<0.0001
208	288 ± 9^bc^	271 ± 10^c^	305 ± 18^b^	416 ± 16^a^	279 ± 10^bc^	<0.0001
Specific growth rate (%/day)*						
0–25	8.95 ± 0.17^ab^	7.75 ± 0.18^c^	9.18 ± 0.11^a^	8.68 ± 0.25^b^	8.55 ± 0.26^b^	<0.0001
26–46	6.56 ± 0.11^a^	5.18 ± 0.77^b^	6.44 ± 0.06^a^	6.48 ± 0.07^a^	6.34 ± 0.10^a^	0.0002
47–70	4.49 ± 0.09	4.34 ± 0.49	4.66 ± 0.17	4.41 ± 0.23	4.47 ± 0.17	0.5763
71–98	2.45 ± 0.16^c^	3.73 ± 0.38^a^	2.47 ± 0.12^c^	3.10 ± 0.12^b^	2.79 ± 0.20^bc^	<0.0001
99–126	1.18 ± 0.12^b^	1.77 ± 0.39^a^	1.19 ± 0.13^b^	1.75 ± 0.14^a^	1.26 ± 0.03^b^	0.0006
127–154	0.53 ± 0.06^ab^	0.77 ± 0.22^a^	0.50 ± 0.07^b^	0.76 ± 0.05^ab^	0.59 ± 0.14^ab^	0.0183
155–182	0.61 ± 0.03	0.62 ± 0.19	0.60 ± 0.04	0.75 ± 0.09	0.56 ± 0.04	0.1412
183–208	0.27 ± 0.08^ab^	0.16 ± 0.09^b^	0.22 ± 0.023^ab^	0.34 ± 0.04^a^	0.22 ± 0.04^ab^	0.0092
Feed intake (% body weight/day)^†^						
26–46	5.96 ± 0.26^ab^	5.91 ± 0.18^ab^	5.79 ± 0.12^b^	6.25 ± 0.23^a^	6.05 ± 0.09^ab^	0.0332
47–70	6.30 ± 0.11	6.23 ± 0.21	6.20 ± 0.07	6.22 ± 0.13	6.21 ± 0.21	0.2873
71–98	5.23 ± 0.15^ab^	5.61 ± 0.20^a^	4.98 ± 0.28^b^	5.42 ± 0.03^a^	5.51 ± 0.18^a^	0.0021
99–126	4.28 ± 0.19^a^	4.56 ± 0.44^a^	3.74 ± 0.05^b^	4.08 ± 0.04^ab^	4.36 ± 0.10^a^	0.0013
127–154	3.53 ± 0.09^a^	3.56 ± 0.36^a^	2.95 ± 0.06^b^	2.90 ± 0.07^b^	3.54 ± 0.15^a^	<0.0001
155–182	3.68 ± 0.16^a^	3.54 ± 0.25^a^	3.06 ± 0.11^b^	2.72 ± 0.14^b^	3.65 ± 0.17^a^	<0.0001
183–208	2.75 ± 0.13^a^	2.81 ± 0.19^a^	2.40 ± 0.06^b^	1.93 ± 0.08^c^	2.82 ± 0.19^a^	<0.0001
Feed efficiency ratio^‡^						
26–46	1.06 ± 0.05^a^	0.88 ± 0.11^b^	1.07 ± 0.02^a^	1.00 ± 0.04^ab^	1.02 ± 0.02^a^	0.0024
47–70	0.79 ± 0.03	0.78 ± 0.06	0.83 ± 0.02	0.79 ± 0.04	0.80 ± 0.05	0.4695
71–98	0.47 ± 0.02^c^	0.63 ± 0.03^a^	0.50 ± 0.03^c^	0.56 ± 0.02^b^	0.50 ± 0.03^bc^	<0.0001
99–126	0.28 ± 0.02^c^	0.39 ± 0.05^ab^	0.33 ± 0.04^bc^	0.44 ± 0.03^a^	0.30 ± 0.01^c^	<0.0001
127–154	0.17 ± 0.02^b^	0.24 ± 0.04^ab^	0.19 ± 0.03^b^	0.29 ± 0.02^a^	0.19 ± 0.04^b^	0.0005
155–182	0.18 ± 0.02^b^	0.19 ± 0.05^b^	0.22 ± 0.02^b^	0.31 ± 0.03^a^	0.17 ± 0.02^b^	0.0003
183–208	0.11 ± 0.03^b^	0.07 ± 0.04^b^	0.11 ± 0.02^b^	0.21 ± 0.02^a^	0.09 ± 0.02^b^	<0.0001

* Specific growth rate (%/day) = 100 × (ln final average weight – ln initial average weight)/days. The specific growth rate of *cck1r*^−/−^ was significantly lower than that of WT during early juvenile stages (0–25 dph, *P* < 0.0001; 26–46 dph, *P* = 0.0005) but significantly higher during later stages (71–98 dph, *P* < 0.0001; 99–126 dph, *P* = 0.0063). The specific growth rate of *cck2rb*^−/−^ was higher than that of WT at 71–126 dph (71–98 dph, *P* = 0.0064; 99–126 dph, *P* = 0.0078).

^†^ Feed intake (% body weight/day) = 100 × food intake/(fish number × (initial BW + final BW) × 0.5 × feeding days). The feed intake in *cck2rb*^−/−^ was lower than that of WT from 127 dph to the end of the trial (127–154 dph, *P* = 0.0018; 155–182 dph, *P* < 0.0001; 183–208 dph, *P* < 0.0001). *cck2ra*^−/−^ exhibited lower feed intake compared to WT at 99–126 dph (*P* = 0.0285), 127–154 dph (*P* = 0.0039), 155–182 dph (*P* = 0.0010), and 183–208 dph (*P* = 0.0224).

^‡^ Feed efficiency ratio = (final total body weight – initial total body weight)/food intake. The feed efficiency ratio of *cck1r*^−/−^ were significantly lower than that of WT during early juvenile stages (26–46 dph, *P* = 0.0044) but significantly higher during later stages (71–98 dph, *P* < 0.0001; 99–126 dph, *P* = 0.0020). The feed efficiency ratio of the *cck2rb*^−/−^ was consistently higher than that of WT throughout the later period (71–98 dph, *P* = 0.0024; 99–126 dph, *P* < 0.0001; 127–154 dph, *P* = 0.0007; 155–182 dph, *P* = 0.0028; 183–208 dph, *P* = 0.0014).

**Table 3:** Body weight (A) and standard length (B) of *cck *receptor KO lines reared for 208 days. Data are presented as mean ± SD (*n* = 20 individuals for 46 and 70 dph; *n* = 8 individuals for 98–208 dph). Values with different letters are significantly different (*P* < 0.05). Standard length and weight of randomly selected five or two individuals per tank were measured (*n* = 20 fish/line at 46 and 70 dph; *n* = 8 fish/sex/line at 98–208 dph).

Parameter/dph/sex	WT	*c* *ck1r* ^−/−^	*c* *ck2ra* ^−/−^	*cck2rb* ^−/−^	*cck2rb* ^+/−^	*P*
Body weight (mg)*						
46 dph						
Male/female	29.0 ± 8.8^a^	17.9 ± 7.8^b^	31.3 ± 8.5^a^	29.4 ± 5.1^a^	27.2 ± 8.0^a^	<0.0001
70 dph						
Male/female	78.0 ± 16.4^ab^	51.3 ± 17.8^c^	83.0 ± 13.6^a^	76.5 ± 18.3^ab^	67.9 ± 18.6^b^	<0.0001
98 dph						
Male	162.7 ± 22.2^a^	131.3 ± 30.8^b^	160.5 ± 11.0^ab^	169.3 ± 18.6^a^	143.1 ± 12.3^ab^	0.0036
Female	153.3 ± 17.9	138.9 ± 30.3	155.2 ± 21.3	173.0 ± 20.8	156.2 ± 24.0	0.0934
126 dph						
Male	187.4 ± 17.7^b^	174.1 ± 14.9^b^	205.7 ± 14.5^ab^	240.0 ± 52.1^a^	198.7 ± 14.2^b^	0.0004
Female	224.1 ± 16.2^b^	216.3 ± 38.2^b^	223.3 ± 26.4^b^	268.1 ± 29.4^a^	224.4 ± 279^b^	0.0062
154 dph						
Male	213.6 ± 20.7^b^	211.1 ± 14.7^b^	228.0 ± 18.1^b^	310.9 ± 37.2^a^	201.0 ± 29.2^b^	<0.0001
Female	245.9 ± 22.7^b^	251.3 ± 38.2^b^	275.8 ± 18.8^b^	348.4 ± 27.0^a^	250.5 ± 40.4^b^	<0.0001
182 dph						
Male	237.7 ± 16.1^bc^	219.3 ± 12.6^c^	262.7 ± 23.3^b^	371.2 ± 40.7^a^	250.1 ± 15.5^bc^	<0.0001
Female	293.8 ± 29.4^b^	317.8 ± 51.1^b^	345.3 ± 57.3^b^	444.9 ± 28.6^a^	292.4 ± 23.6^b^	<0.0001
208 dph						
Male	246.7 ± 16.1^bc^	228.2 ± 19.2^c^	286.9 ± 37.4^b^	389.4 ± 40.0^a^	248.9 ± 34.9^bc^	<0.0001
Female	350.8 ± 45.5^bc^	366.4 ± 65.5^bc^	369.6 ± 60.5^b^	462.7 ± 55.7^a^	292.6 ± 28.0^c^	<0.0001
Standard length (mm)						
46 dph						
Male/female	12.6 ± 1.2^a^	10.9 ± 1.5^b^	12.9 ± 1.2^a^	12.9 ± 0.7^a^	12.4 ± 1.2^a^	<0.0001
70 dph						
Male/female	17.0 ± 1.2^ab^	17.0 ± 1.8^c^	17.6 ± 0.9^a^	17.1 ± 1.3^ab^	16.4 ± 1.4^b^	<0.0001
98 dph						
Male	21.7 ± 0.8^ab^	20.4 ± 1.6^b^	21.9 ± 0.6^a^	22.1 ± 0.9^a^	21.1 ± 0.5^ab^	0.0065
Female	21.3 ± 0.8	20.3 ± 1.5	21.1 ± 1.2	22.1 ± 1.1	21.1 ± 1.2	0.0995
126 dph						
Male	22.7 ± 0.3^bc^	22.1 ± 0.9^c^	23.6 ± 0.6^ab^	24.3 ± 1.6^a^	23.4 ± 0.6^ab^	0.0003
Female	23.7 ± 1.0^ab^	23.2 ± 1.1^b^	23.7 ± 1.0^ab^	25.0 ± 1.0^a^	23.7 ± 0.8^ab^	0.0106
154 dph						
Male	24.3 ± 1.1^b^	24.4 ± 0.7^b^	24.5 ± 0.6^b^	26.7 ± 1.0^a^	23.8 ± 1.3^b^	<0.0001
Female	24.9 ± 0.8^b^	24.3 ± 1.2^b^	25.7 ± 0.38^b^	27.6 ± 1.1^a^	24.5 ± 1.2^b^	<0.0001
182 dph						
Male	25.2 ± 0.8^b^	24.7 ± 1.0^b^	25.8 ± 0.7^b^	28.1 ± 1.2^a^	25.6 ± 0.5^b^	<0.0001
Female	26.5 ± 1.1^b^	26.5 ± 1.0^b^	27.9 ± 1.4^b^	29.6 ± 1.1^a^	26.4 ± 0.8^b^	<0.0001
208 dph						
Male	25.6 ± 0.5^bc^	25.0 ± 1.2^c^	26.8 ± 1.2^b^	28.6 ± 0.9^a^	25.6 ± 1.1^bc^	<0.0001
Female	28.1 ± 1.3^b^	27.5 ± 1.4^b^	28.2 ± 1.2^b^	30.1 ± 0.7^a^	26.9 ± 1.3^b^	<0.0001
Condition factor^†^						
46 dph						
Male/female	14.1 ± 0.2^ab^	13.1 ± 0.3^c^	14.5 ± 0.3^a^	13.6 ± 0.2^bc^	13.8 ± 0.2^abc^	0.0003
70 dph						
Male/female	15.5 ± 0.2^a^	14.5 ± 0.2^b^	15.0 ± 0.2^ab^	15.0 ± 0.2^ab^	15.0 ± 0.3^ab^	0.0227
98 dph						
Male	15.9 ± 0.5	15.3 ± 0.2	15.3 ± 0.2	15.7 ± 0.2	15.1 ± 0.2	0.4968
Female	15.9 ± 0.2	16.2 ± 0.4	16.4 ± 0.3	16.1 ± 0.4	16.5 ± 0.4	0.3822
126 dph						
Male	15.9 ± 0.4	16.2 ± 0.4	15.6 ± 0.4	16.5 ± 0.4	15.5 ± 0.2	0.3658
Female	16.9 ± 0.5	17.2 ± 0.5	16.8 ± 0.5	17.1 ± 0.3	16.8 ± 0.5	0.9435
154						
Male	15.0 ± 0.5^ab^	14.6 ± 0.3^b^	15.5 ± 0.4^ab^	16.2 ± 0.3^a^	14.8 ± 0.3^ab^	0.0312
Female	16.0 ± 1.0	17.5 ± 0.6	16.3 ± 0.3	16.5 ± 0.3	17.0 ± 0.7	0.1992
182 dph						
Male	14.9 ± 0.4^b^	14.7 ± 0.5^b^	15.3 ± 0.3^ab^	16.7 ± 0.4^a^	15.0 ± 0.3^b^	0.0076
Female	15.8 ± 0.4	16.9 ± 0.5	15.8 ± 0.3	17.2 ± 0.5	15.9 ± 0.2	0.0273
208 dph						
Male	14.8 ± 0.4^b^	14.7 ± 0.4^b^	14.8 ± 0.2^b^	16.7 ± 0.4^a^	14.7 ± 0.2^b^	0.0003
Female	15.7 ± 0.3^ab^	17.6 ± 0.9^a^	16.4 ± 0.5^ab^	17.0 ± 0.5^b^	15.1 ± 0.4^ab^	0.0317

* An increased body weight in both male and female *cck2rb*^−/−^ was observed from 126 dph to the end of the trial (126 dph: male, *P* = 0.0037, female, *P* = 0.0297; 154 dph: male, *P* < 0.0001, female, *P* < 0.0001; 182 dph: male, *P* < 0.0001, female, *P* < 0.0001; 208 dph: male, *P* < 0.0001, female, *P* = 0.0014). A greater standard length of both sexes of *cck2rb*^−/−^ was also observed from 126 dph onward, except for females at 126 dph (126 dph: male, *P* = 0.0128, female, *P* = 0.0770; 154 dph: male, *P* = 0.0002, female, *P* < 0.0001; 182 dph: male, *P* < 0.0001, female, *P* < 0.0001; 208 dph: male, *P* < 0.0001, female, *P* = 0.0214).

^†^ Condition factor = 1,000 × body weight (mg)/standard length (mm)^3^. The condition factor of *cck1r*^−/−^ was lower than that of WT at 46 (*P* = 0.0124) and 70 dph (*P* = 0.0078). Male *cck2rb*^−/−^ exhibited a significantly higher condition factor than the other lines at 182 (*P* = 0.0205) and 208 dph (*P* = 0.0023).

### Reproductive performance in females

First spawning was observed at 78 dph in WT, 81 dph in *cck2rb*^+/−^, 85 dph in *cck2ra*^−/−^, and 86 dph in *cck1r*^−/−^ whereas *cck2rb*^−/−^ did not spawn any eggs during the trial. Total egg number/female (including non-spawning females in the calculation) in *cck2ra*^−/−^ was significantly lower than in WT, with *cck1r*^−/−^ showing the lowest values among the lines (Fig. 3B). Total egg number/spawned female in *cck2ra*^−/−^ was equivalent to WT, while *cck1r*^−/−^ females produced fewer eggs than the other lines (Fig. 3C). The total egg number/female of *cck2rb*^+/−^ was significantly lower than that of WT, whereas total egg number/spawned female was comparable to that of WT, indicating lower spawning frequency of *cck2rb*^+/−^ females.

### Internal organ and plasma vitellogenin

**Figure 4:**
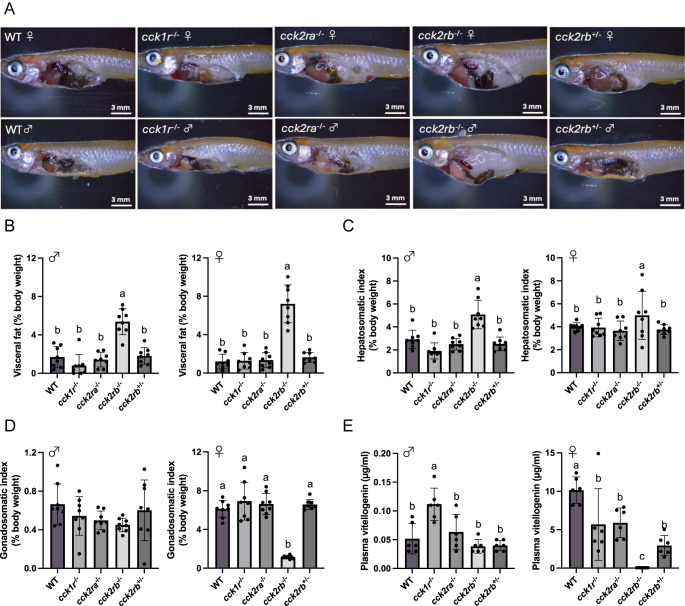
Internal organ and plasma vitellogenin in male and female *cckr* KO medaka. Values with different letters are significantly different (*n* = 8 fish, *P* < 0.05). (A) Anatomical observations for representative images of WT and *cckr* mutant lines. Both male and female *cck2rb*^−/−^ medaka exhibited pronounced visceral fat accumulation. Scale bar: 3 mm. (B) Visceral fat ratio of the *cck2rb*^−/−^ line was the highest in both male and female (*P* < 0.0001). (C) The hepatosomatic index of male *cck2rb*^−/−^ was significantly higher than that of the other lines (*P* < 0.0001), while there were no significant differences in females (*P* = 0.3945). (D) The female *cck2rb*^−/−^ gonadosomatic index was low compared to those of the other lines (*P* < 0.0001). (E) Plasma vitellogenin levels were significantly elevated in male *cck1r*^−/−^ compared to WT (*P* = 0.0011). In females, plasma vitellogenin levels were reduced in all *cckr* KO lines compared to WT, with *cck2rb*^−/−^ showing levels near to the detection limit (*cck1r*^−/−^ vs WT, *P* = 0.0308; *cck2rb*^−/−^ vs WT, *P* < 0.0001; *cck2rb*^+/−^ vs WT, *P* = 0.0003; *cck2ra*^−/−^ vs WT, *P* = 0.0437). A full color version of this figure is available at https://doi.org/10.1530/JOE-25-0353.

To investigate how each Cckr KO line affects fat accumulation and sexual maturation, we analyzed visceral fat ratio, gonadosomatic index, and plasma vitellogenin levels. The visceral fat ratio of the *cck2rb*^−/−^ was the highest in both males and females (Fig. 4A and B). The hepatosomatic index of male *cck2rb*^−/−^ was significantly higher than that of the other lines, while no significant differences were observed in females (Fig. 4C).

No differences were found in male gonadosomatic index (Fig. 4D). The female *cck2rb*^−/−^ gonadosomatic index was markedly lower compared to those of the other lines. Although plasma vitellogenin levels were significantly elevated in male *cck1r*^−/−^, the absolute concentrations remained much lower than those observed in females, reaching only 1% of the female values (Fig. 4E). In females, plasma vitellogenin levels were reduced in all *cckr* KO lines compared to WT, with *cck2rb*^−/−^ showing levels close to the detection limit.

### Lipid composition

**Figure 5:**
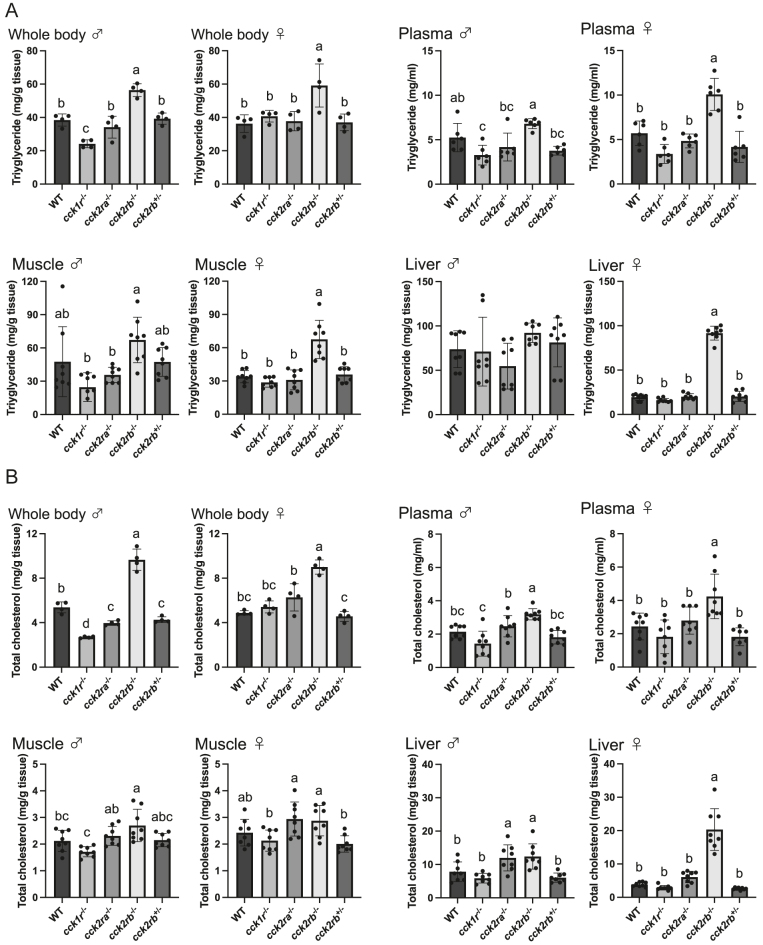
Lipid compositions of *cckr* KO medaka. Values with different letters are significantly different (*n* = 4 pooled samples for the whole body; *n* = 8 fish for the plasma, muscle, and liver, *P* < 0.05). (A) The *cck2rb*^−/−^ line exhibited significantly elevated whole-body triglyceride levels in males (*P* = 0.0002) and females (*P* = 0.0037), as well as in female plasma (*P* = 0.0001) and female muscle (*P* < 0.0001), compared to WT. Liver triglyceride levels of female *cck2rb*^−/−^ were markedly higher compared to the other lines (*P* < 0.0001). Triglyceride levels in *cck1r*^−/−^ males were significantly lower than those in WT in the whole body (*P* = 0.0018) and plasma (*P* = 0.0493). (B) Total cholesterol levels were significantly elevated in *cck2rb*^−/−^ in both male and female whole body (*P* < 0.0001), male and female plasma (male, *P* = 0.0028; female, *P* = 0.0042), male muscle (*P* = 0.0399), and especially in the female liver (male, *P* = 0.0263; female, *P* < 0.0001). Whole-body total cholesterol levels in *cck1r*^−/−^ males were significantly lower than those in WT (*P* < 0.0001).

Given the observed fat accumulation in the *cckr* KO lines, lipid compositions in the whole body, plasma, muscle, and liver were analyzed (Fig. 5). The *cck2rb*^−/−^ showed significantly elevated triglyceride levels in both sexes in the whole body, as well as in female plasma and muscle, compared to WT. While no difference was observed in male liver triglycerides, female *cck2rb*^−/−^ livers exhibited levels over four times higher than WT livers. In contrast, *cck1r*^−/−^ males had significantly lower triglyceride levels in the whole body and plasma. Similarly, total cholesterol levels were significantly increased in *cck2rb*^−/−^ males and females across the whole body, plasma, muscle, and especially in the female liver. Conversely, *cck1r*^−/−^ males showed significantly reduced whole-body cholesterol levels compared to WT.

### Digestive enzymes secretion

**Figure 6:**
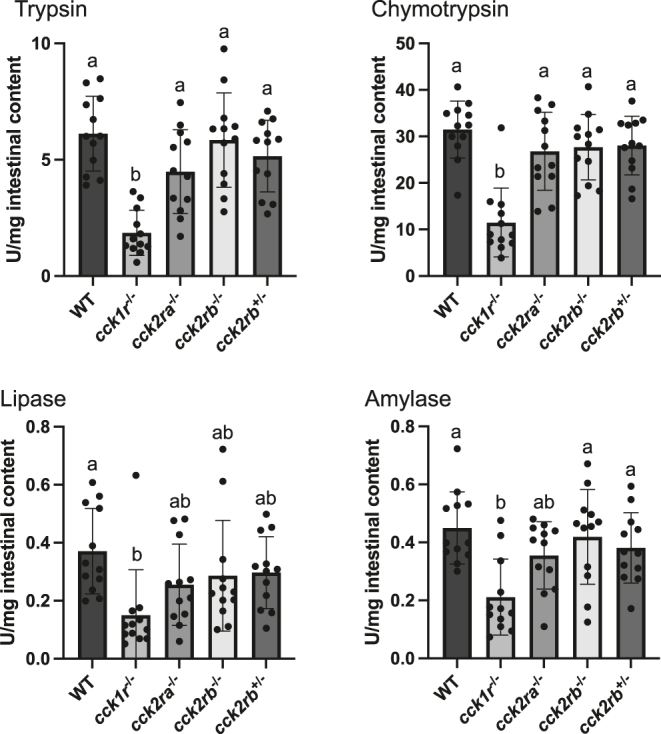
Secretion levels of digestive enzymes in *cckr* KO medaka. Values with different letters are significantly different (*n* = 12 fish; results of six male and six female were combined). The activities were defined as the amount of *p*-nitroaniline (nmol), *p*-nitrophenol (nmol), or maltose (μmol) released from the substrate used (N-benzoyl-L-Arg-*p*-nitroanilide for trypsin, N-succinyl-Ala-Ala-Pro-Phe *p*-nitroanilide for chymotrypsin, *p*-nitrophenyl myristate for lipase, and starch for amylase) in 1 min. All the enzymes exhibited lower secretion levels in *cck1r*^−/−^ compared to WT (trypsin, *P* < 0.0001; chymotrypsin, *P* < 0.0001; lipase, *P* = 0.0073; amylase, *P* = 0.0004).

To evaluate how each *cckr* KO line affects pancreatic exocrine function, the activities of four digestive enzymes in the intestinal contents were quantified (Fig. 6). All enzyme activities were significantly reduced in *cck1r*^−/−^, with trypsin and chymotrypsin activities reduced to approximately 30% of WT levels. Lipase and amylase activities were also significantly reduced, although the decreases were less pronounced than those observed for proteases.

### Gene expression

**Figure 7:**
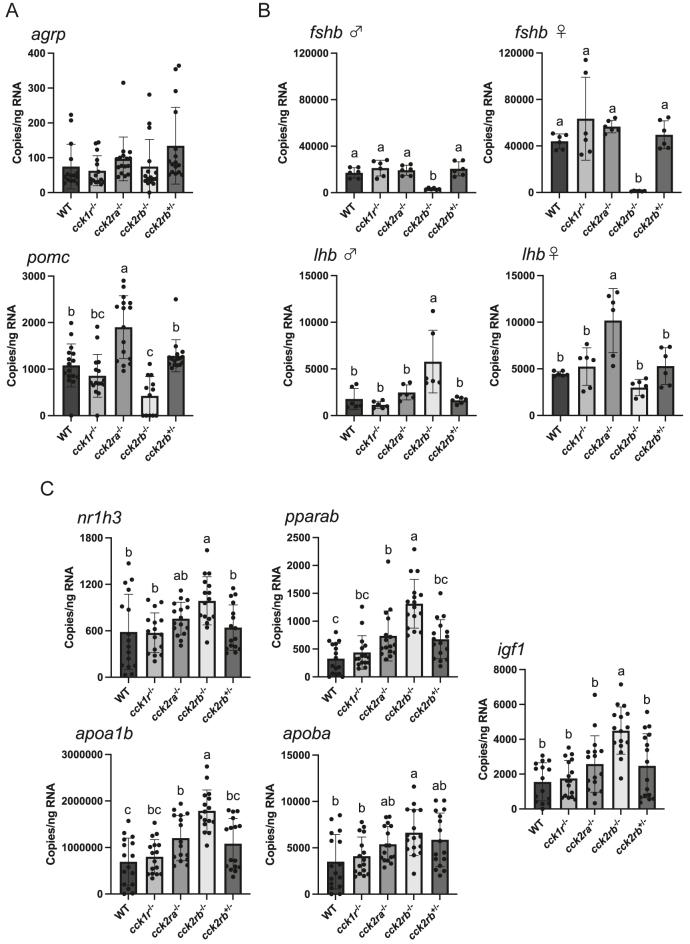
Expression levels of appetite-, sex maturation-, lipid metabolism-, and growth-related genes in cckr KO medaka. (A) Expression levels of hypothalamic appetite-related genes. Values with different letters are significantly different (*n* = 16 fish, results of eight male and eight female were combined, *P* < 0.05). The levels of *pomc* was higher in the *cck2ra*^−/−^ (*P* < 0.0001) and lower in the *cck2rb*^−/−^ (*P* = 0.0068) than that of WT. (B) Expression levels of pituitary gonadotropin hormones. Values with different letters are significantly different (*n* = 6, pooled from 3 fish each; 18 fish/sex/line, *P* < 0.05). The expression level of *fshb* in *cck2rb*^−/−^ line was lower than that of WT in both males (*P* = 0.0005) and females (*P* = 0.0019). The *lhb* expression was significantly higher in male *cck2rb*^−/−^ (*P* = 0.0023) and female *cck2ra*^−/−^ (*P* = 0.0005) compared to those in WT, respectively. (C) Expression levels of lipid metabolism- and growth-related genes in the liver. Values with different letters are significantly different (*n* = 16 fish, results of eight male and eight female were combined, *P* < 0.05). Expression levels of lipid metabolism related-genes, *nr1h3* (*P* = 0.0071), *pparab* (*P* = 0.0005), *apoa1b* (*P* < 0.0001), and *apoba* (*P* = 0.0059) in *cck2rb*^−/−^, were higher than those in WT. Significantly higher expression levels of *pparab* (*P* = 0.0205) and *apoa1b* (*P* = 0.0272) were observed in *cck2ra*^−/−^ compared to WT. The expression level of *igf1* in *cck2rb*^−/−^ was higher than that of WT (*P* < 0.0001).

Cck is known to regulate appetite and sexual maturation. The hypothalamic expression of the orexigenic factor *agrp* remained unchanged, while the anorexigenic factor *pomc* was upregulated in *cck2ra*^−/−^ and downregulated in *cck2rb*^−/−^ compared to WT (Fig. 7A). The expression level of pituitary gonadotropin hormone *fshb* in *cck2rb*^−/−^ was lower than that of WT in both males and females (Fig. 7B). The *lhb* expression was significantly higher in male *cck2rb*^−/−^ and female *cck2ra*^−/−^ compared to those in WT, respectively.

Novel Cck-related phenotypes were identified in lipid metabolism and growth. The hepatic expression of lipid metabolism-related genes, *nr1h3*, *pparab*, *apoa1b*, and *apoba*, was elevated in *cck2rb*^−/−^ compared to WT (Fig. 7C). The genes *pparab* and *apoa1b* were also significantly upregulated in *cck2ra*^−/−^. Moreover, the hepatic expression of the growth-promoting gene *igf1* was higher in *cck2rb*^−/−^ than in WT.

### Histological observation

**Figure 8:**
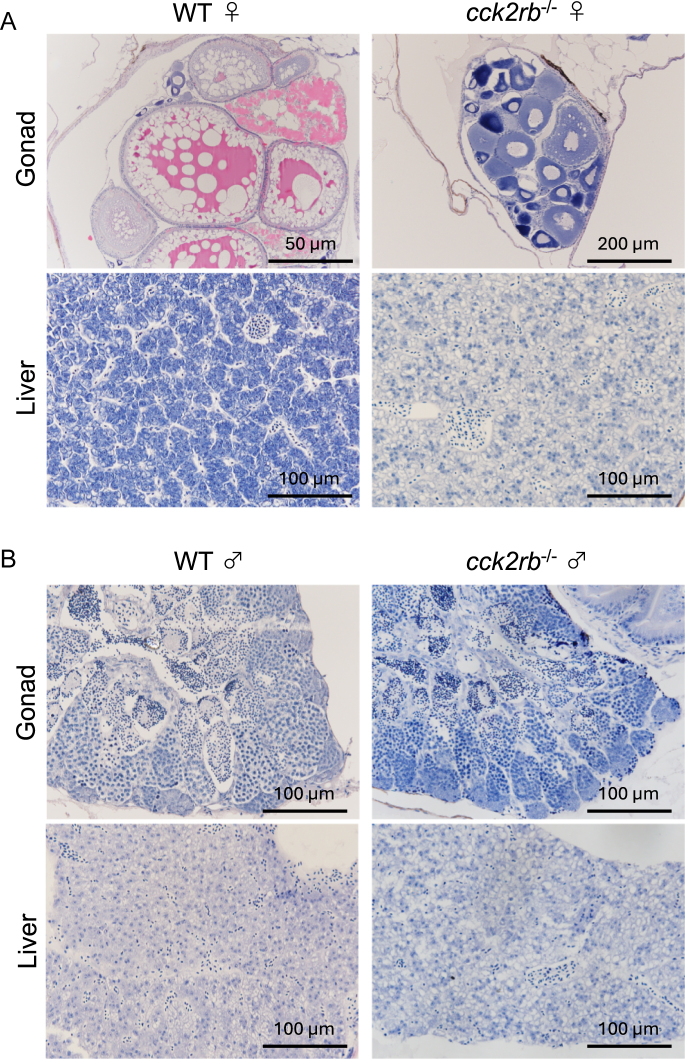
Histological observations of gonads and liver in WT and *cck2rb*^−/−^ mutant. (A) Representative sections of ovaries and liver of WT and *cck2rb*^−/−^ female line. (B) Representative sections of testes and liver from WT and *cck2rb*^−/−^ male line. Scale bar: 50, 100, or 200 μm. A full color version of this figure is available at https://doi.org/10.1530/JOE-25-0353.

Histological analysis revealed distinct differences in gonadal development and liver morphology between WT and *cck2rb*^−/−^. In WT females, fully developed oocytes were observed in the ovaries, whereas previtellogenic oocytes were present in *cck2rb*^−/−^ females (Fig. 8A). In contrast, spermatogenesis proceeded in both *cck2rb*^−/−^ and WT testes, with spermatozoa filling the lumen of the sperm ducts (Fig. 8B); however, potential differences in the spermatogenic process cannot be excluded without additional studies. In *cck2rb*^−/−^ females, the liver showed a steatosis-like morphology with numerous vacuoles resembling oil droplets, whereas males displayed no clear differences from WT (Fig. 8A and B). No discernible morphological differences were observed in gonads or liver among WT, *cck1r*^−/−^, and *cck2ra*^−/−^ (data not shown).

### Transcriptomic analysis

**Figure 9:**
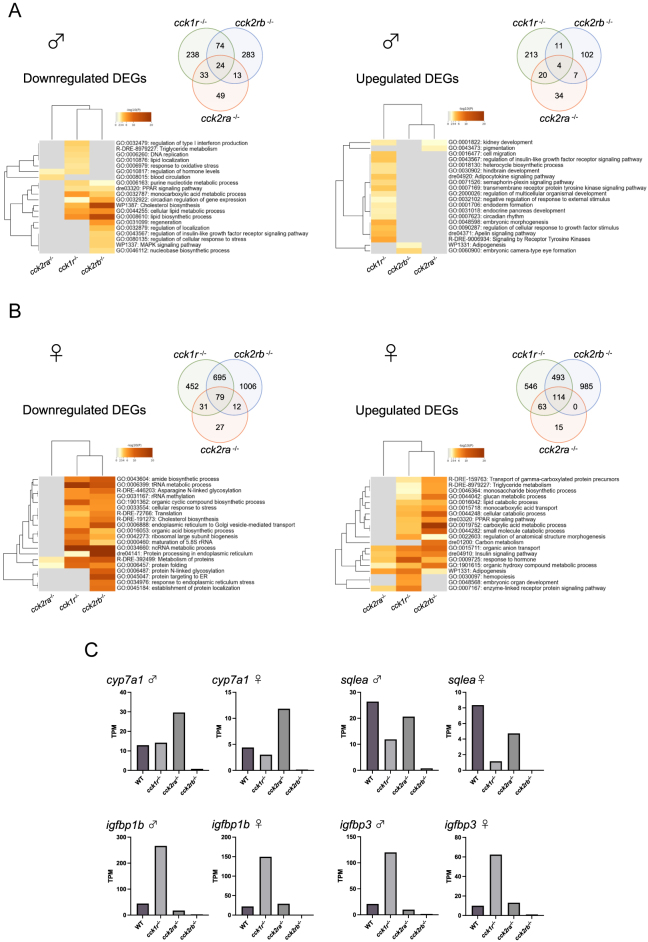
Differentially expressed genes (DEGs) identified in *cckr* KO medaka comparison with the WT. Following the removal of low-quality reads, adaptor sequences, and reads containing a high proportion of unknown bases, clean reads with the following average read counts were obtained: WT, 12.1 million for male and 12.1 million for female; *cck1r*^−/−^, 11.7 million for male and 11.9 million for female; *cck2ra*^−/−^, 12.0 million for male and 12.1 million for female; and *cck2rb*^−/−^, 11.7 million for male and 12.1 million for female. The RNA-seq read datasets generated in this study were deposited in the DDBJ sequence read archive under accession numbers DRR720458–DRR720497. Approximately 93.71% of the RNA-seq reads were mapped on the reference genome. (A and B) Numbers of upregulated and downregulated DEGs in each group are shown in Venn diagrams of the DEGs across the *cck1r*^−/−^, *cck2ra*^−/−^, and *cck2rb*^−/−^ lines. Heat maps show the enriched GO terms (biological processes) in downregulated and upregulated DEGs of each group. The bars are colored according to the *P*-value, with darker colors indicating lower *P*-values. (C) Expression of genes showing differences identified in the transcriptomic analysis. Gene expression levels were calculated as TPM values. A full color version of this figure is available at https://doi.org/10.1530/JOE-25-0353.

Transcriptomic analyses of liver tissues were performed in *cckr* KO lines to investigate the mechanisms underlying the novel metabolic and growth-related phenotypes. In males, 617, 184, and 518 DEGs were identified in *cck1r*^−/−^, *cck2ra*^−/−^, and *cck2rb*^−/−^, respectively, compared to WT (Fig. 9A). GO enrichment analysis showed downregulation of genes involved in cholesterol and lipid biosynthesis in *cck1r*^−/−^ and *cck2rb*^−/−^ males, while *cck1r*^−/−^ males also exhibited upregulation of genes associated with the Igf signaling pathway.

In females, 2,473, 341, and 3,384 DEGs were found in *cck1r*^−/−^, *cck2ra*^−/−^, and *cck2rb*^−/−^, respectively (Fig. 9B). Similar to males, cholesterol biosynthesis genes were downregulated in *cck1r*^−/−^ and *cck2rb*^−/−^ females. Notably, *cck2rb*^−/−^ females showed upregulation of genes associated with triglyceride metabolism, lipid catabolism, and the PPAR signaling pathway. Adipogenesis-related genes were upregulated across all KO lines.

The expression profiles of selected genes within enriched pathways were visualized using transcripts per million (TPM) values (Fig. 9C). Cytochrome P450 family 7 subfamily A member 1 (*cyp7a1*) showed consistent sex-independent expression patterns with reduced expression in *cck2rb*^−/−^ and increased expression in *cck2ra*^−/−^. Across sexes, insulin-like growth factor-binding proteins 1b and 3 (*igfbp1b* and *igfbp3*) were upregulated in *cck1r*^−/−^ and downregulated in *cck2rb*^−/−^, whereas squalene epoxidase a (*sqlea*) was downregulated in both *cck1r*^−/−^ and *cck2ra*^−/−^.

## Discussion

In the present study, we successfully generated three distinct Cck receptor KO medaka: *cck1r*^−/−^, *cck2ra*^−/−^, and *cck2rb*^−/−^. The most prominent phenotype observed was female infertility in *cck2rb*^−/−^. This finding is consistent with a recent report by Uehara *et al.* (14), which identified a novel Cck signaling pathway in medaka, wherein hypothalamic-derived Cck stimulates Fsh release from the pituitary via activation of Cck2rb. In that study, knockout of Cck2rb or its ligand resulted in female infertility.

Fsh cells express *cck2r* paralogs in teleosts, including *cck2rb* in tilapia (21), *cck2ra* in zebrafish (22), and *cck2rb* in medaka (14), showing a direct function in reproductive maturation. This is consistent with our results showing that pituitary is the primary site of *cck2rb* expression, and we further confirmed reduced *fshb* expression in the pituitary of *cck2rb*^−/−^. Additionally, the immature ovaries and reduced gonadosomatic index in female *cck2rb*^−/−^ suggest estrogen deficiency, consistent with Uehara *et al.* (14). However, unlike their report, where *lhb* expression was lower in females and unchanged in males, our study found it unchanged in females but slightly increased in males. Uehara *et al.* (14) also reported a markedly reduced gonadosomatic index in *cck2rb*^−/−^ males, whereas no difference was observed in our study, which may be related to differences in age and body size between the fish used, as their report examined smaller, younger fish compared to ours.

Plasma vitellogenin levels, a marker of female reproductive maturity in fish (23), were also lower in *cck1r*^−/−^ and *cck2ra*^−/−^ compared to WT. Onset of spawning was delayed in *cck1r*^−/−^ and *cck2ra*^−/−^, and their total egg number per female was lower than in WT. This suggests that Cck1r and Cck2ra may contribute to FSH regulation, but whether their effects are direct or mediated indirectly through factors such as growth, feeding, or metabolism remains unclear. As for the function of Cck2ra in other fish species, Hollander-Cohen *et al.* (22) showed that Cck2ra KO zebrafish develop exclusively as males with underdeveloped gonads, underscoring possible species-specific differences in sex determination and maturation.

Control of digestion is one of the well-known Cck functions. CCK plays a pivotal role in stimulating the exocrine secretion of the pancreas and promoting bile release from the gallbladder (24, 25). In fish, administration of mammalian CCK stimulates the secretion of trypsin and chymotrypsin and gallbladder discharge (26). In fish, *cck1r* is highly expressed in the gastrointestinal tract and is therefore considered to be primarily involved in digestion (27). This study also confirmed that medaka *cck1r* is highly expressed in digestion-related tissues, including the gastrointestinal tract and gallbladder. Moreover, secretion levels of pancreatic digestive enzymes, trypsin, chymotrypsin, lipase, and amylase were significantly lower in *cck1r*^−/−^ than those in the other fish lines. In teleosts, the role of *cck1r* in digestive enzyme secretion has only been inferred from indirect evidence, such as tissue expression patterns. Thus, our study provides the first direct demonstration of this function. Such reduced digestive enzyme secretion in *cck1r*^−/−^ may impair their feed utilization and subsequent growth performance. Indeed, reduced feed efficiency and significant growth retardation were observed in *cck1r*^−/−^, with fish weighing only 56% of WT at the juvenile stage.

Conversely, *cck2rb*^−/−^ line exhibited significantly greater growth performance at the post-juvenile stage compared to the other lines. While Uehara *et al.* (14) reported no detectable growth differences in *cck2rb*^−/−^, our study employed a more detailed long-term rearing design with multiple replicates and regular measurements, which may have enabled identification of this phenotype. Infertile fish, such as triploids, often show enhanced somatic growth due to reduced energy allocation to reproduction (28). However, *cck2rb*^−/−^ exhibited improved growth not only in infertile females but also in fertile males, suggesting a direct role of Cck in growth regulation. This was supported by elevated hepatic *igf1* expression, a key growth-promoting factor in vertebrates (29). Considering that *cck2rb* is predominantly expressed in the pituitary, it is plausible that Cck signaling influences the brain–pituitary–growth axis through endocrine pathways. However, it should be noted that Uehara *et al.* (14) reported a smaller testis size in males, indicating that an imbalance in energy allocation cannot be completely ruled out. Integration of single-cell transcriptomic data would provide valuable insights into pituitary cell-type specificity of Cck receptor expression (30), which is an important direction for future research.

Besides its role in digestion, Cck functions as a potent anorexigenic signal in vertebrates, including fish (31). In mammals, peripheral CCK induces satiety via CCK1 receptors on vagal afferents, providing feedback to brain (32). A similar vagus-mediated mechanism has been proposed in fish (33). In this study, *pomc*, a central appetite-suppressing gene, was significantly downregulated in the *cck2rb*^−/−^, suggesting a potential involvement of Cck2rb in appetite regulation. However, no clear differences in feed intake were detected among the Cckr KO lines. Given that Cck acts as a short-term satiety factor in rainbow trout (34), the lack of effect may reflect limitations of the KO approach under the experimental conditions. Alternatively, redundant gut–brain signaling may compensate through other neural or peptide pathways (35).

In addition to roles in maturation, digestion, and appetite regulation, our study revealed a striking lipid metabolic phenotype in Cckr KO medaka. Both male and female *cck2rb*^−/−^ individuals exhibited markedly elevated whole-body lipid levels, including increased accumulation of visceral fat, triglycerides, and cholesterol, indicating a pronounced phenotype associated with disrupted lipid homeostasis. Although excessive lipids are usually deposited around the abdominal viscera in medaka (36), *cck2rb*^−/−^ also showed elevated triglyceride and cholesterol levels in plasma, muscle, and liver, in addition to the abdominal viscera. Histological examination further confirmed hepatic lipid accumulation in *cck2rb*^−/−^ females, indicating disrupted liver lipid metabolism associated with the loss of *cck2rb* function.

The elevated hepatic levels of triglycerides and cholesterol observed in infertile females were particularly pronounced, likely reflecting reduced energy expenditure due to the absence of the reproductive investments of vitellogenesis, maturation, and spawning. In mammals, *NR1H3* is a cholesterol-sensing nuclear receptor that promotes cholesterol efflux and lipid homeostasis (37), and PPARA regulates genes involved in lipid catabolism via hepatic β-oxidation (38). APOA1 and APOB encode major apolipoproteins of HDL and LDL, which mediate reverse and forward cholesterol transport, respectively (39). In this study, the expression levels of these genes were significantly upregulated in the *cck2rb*^−/−^, suggesting an enhancement of the overall lipid metabolic processes. In particular, the concomitant upregulation of *nr1h3* and *ppara* suggests an enhancement of nuclear receptor-mediated signaling pathways, which may represent a compensatory response to lipid accumulation rather than a primary driver of lipid catabolism. Furthermore, the upregulation of apolipoprotein genes suggests increased lipoprotein biosynthesis and remodeling, which may influence intracellular lipid trafficking and systemic lipid distribution (40).

Transcriptomic analysis revealed that both male and female *cck1r*^−/−^ and *cck2rb*^−/−^ exhibited downregulated expression of genes associated with cholesterol biosynthesis, including *sqlea*. While this is consistent with the lower whole-body cholesterol levels observed in *cck1r*^−/−^, *cck2rb*^−/−^ paradoxically exhibited an increase in cholesterol levels. This discrepancy may be attributed to impaired cholesterol metabolism, including the elimination pathways in *cck2rb*^−/−^ medaka. Cholesterol accumulation is determined not only by its biosynthesis but also by the balance among synthesis, metabolism, and efflux (41). Transcriptomic analysis in this study also revealed significant downregulation of *cyp7a1* expression in *cck2rb*^−/−^, suggesting a reduced conversion of cholesterol into bile acids (42). Additionally, in infertile *cck2rb*^−/−^ females, cholesterol may not have been effectively utilized for the biosynthesis of steroid hormones, which are closely associated with reproductive function (43). These findings suggest that cholesterol accumulation may have resulted from impaired regulatory mechanisms rather than increased biosynthesis.

Although cholesterol biosynthetic pathways were downregulated, female *cck1r*^−/−^, *cck2ra*^−/−^, and/or *cck2rb*^−/−^ exhibited upregulated expression of genes associated with triglyceride metabolism, lipid catabolic process, PPAR signaling pathway, and adipogenesis. These transcriptional changes partially correspond to the observed elevation of lipid levels in *cck2rb*^−/−^, further supporting the observation that lipid metabolism is disrupted in *cckr* KO fish.

Furthermore, transcriptomic analysis of male *cck1r*^−/−^ revealed an upregulation of genes associated with the regulation of the Igf signaling pathway, including *igfbp1b* and *igfbp3*. This molecular profile is consistent with the reduced somatic growth observed in *cck1r*^−/−^. Igfbp1b and Igfbp3 are known to limit the Igf signaling and act as negative regulators of growth in teleost (44). Therefore, it is plausible that Cck1r-mediated signaling plays an important role in the regulation of somatic growth in teleost fish.

Compared to *cck1r*^−/−^ and *cck2rb*^−/−^ lines, no apparent phenotypic alterations were observed in the *cck2ra*^−/−^ line in this study. Tissue distribution analysis revealed that *cck2ra* is highly expressed in the pituitary, as with *cck2rb*. In female *cck2ra*^−/−^, reproductive maturation indicators, such as total egg per female and plasma vitellogenin concentration, showed a downward trend compared to WT. Additionally, subtle changes in increased whole-body lipid content and the upregulated lipid metabolism-related genes were detected in *cck2ra*^−/−^ medaka. These findings suggest that Cck2ra signaling is partially involved in the regulation of reproductive maturation and lipid metabolic processes.

In summary, our comprehensive analysis of three distinct Cckr KO lines in medaka highlights the multifaceted physiological functions of Cck signaling in teleosts. The findings not only reaffirm Cck’s established roles in digestion and reproductive maturation but also uncover its contributions to lipid metabolism and somatic growth. Notably, the phenotypic divergence among the receptor subtypes supports the concept of functional specialization: Cck1r primarily mediates digestive enzyme secretion and promotes early growth; Cck2ra appears to contribute to reproductive maturation and lipid metabolism regulation; and Cck2rb regulates lipid metabolism and reproduction, as well as suppresses body size. The pronounced lipid accumulation and enhanced somatic growth observed in *cck2rb*^−/−^ could reflect both direct effects of Cck signaling and indirect consequences of impaired reproduction. Further studies will be important to clarify the relative contributions of these mechanisms, for example by hormonal profiling or ligand administration experiments.

## Declaration of interest

The authors declare that there is no conflict of interest that could be perceived as prejudicing the impartiality of the work reported.

## Funding

This work was supported by JSPS KAKENHI JP22K05826 and JP25K09283. IR, AG, and KM also acknowledge support from the Research Council of Norway (ExcelAQUA 2.0, Grant No. 309368).

## Data availability

Data supporting this study are available on reasonable request.
